# Effects of colistin and tigecycline on multidrug-resistant *Acinetobacter baumannii* biofilms: advantages and disadvantages of their combination

**DOI:** 10.1038/s41598-021-90732-3

**Published:** 2021-06-03

**Authors:** Yoshinori Sato, Tsuneyuki Ubagai, Shigeru Tansho-Nagakawa, Yusuke Yoshino, Yasuo Ono

**Affiliations:** Department of Microbiology and Immunology, Teikyo University School of Medicine, 2-11-1 Kaga, Itabashi-ku, Tokyo, 173-8605 Japan

**Keywords:** Antimicrobials, Bacteria, Biofilms

## Abstract

We investigated the antimicrobial effects of colistin (CST) and tigecycline (TGC), either alone or in combination, on biofilm-dispersed and biofilm-embedded multidrug-resistant *Acinetobacter baumannii* (MDRAB) strains R1 and R2. The bacterial growth of biofilm-dispersed MDRAB was inhibited by CST or TGC. However, the inhibitory effects were attenuated by a combination of CST and low concentrations of TGC. The bactericidal effects of CST, but not TGC, were observed on biofilm-dispersed MDRAB. Notably, the bactericidal effects increased with a combination of CST and high concentrations of TGC, whereas they were attenuated with the combination of CST and low concentrations of TGC. Although biofilm formation by MDRAB decreased with increasing concentrations of CST or TGC, there was no complete disruption of the biofilms. Additionally, the biofilms increased with a combination of 1–2 μg/mL CST and TGC at 2 μg/mL and 2–4 μg/mL for strains R1 and R2, respectively. Biofilm-embedded MDRAB was eradicated with CST, but not TGC. Notably, the eradication effects increased with a combination of CST and high concentrations of TGC, whereas attenuation happened with the combination of CST and low concentrations of TGC. These results provide information on the combined effects of CST and TGC in the treatment of biofilm-associated MDRAB infection.

## Introduction

*Acinetobacter baumannii* is an important opportunistic pathogen associated with nosocomial infections, such as bacteremia, pneumonia, meningitis, urinary tract infections, and wound infections^1,2^. Additionally, *A. baumannii* is included among the six nosocomial pathogens: *Enterococcus faecium*, *Staphylococcus aureus*, *Klebsiella pneumoniae*, *Acinetobacter baumannii*, *Pseudomonas aeruginosa*, and *Enterobacter* spp. (ESKAPE) that acquire multidrug resistance and virulence^3,4^. Therefore, *A. baumannii*, especially multidrug-resistant *A. baumannii* (MDRAB), has gained importance as a human pathogen in hospital environments.

Although *A. baumannii* is regarded as a low-virulence pathogen^5^, recent studies have shown that *A. baumannii* exhibits several forms of pathogenicity, such as biofilm formation, adherence, invasion of lung epithelial cells, host cell death, and iron acquisition^6^. Recently, Colquhoun et al. hypothesized that biofilms are, at least in part, responsible for the high prevalence of *A. baumannii* nosocomial and recurrent infections because they frequently contaminate hospital surfaces and patient indwelling devices^7^. *A. baumannii* is capable of forming biofilms^2,5,6^; biofilm persister cells are protected from antimicrobial agents and immune responses and are therefore difficult to eliminate^8^, suggesting bacterial colonization in hosts and promoting persistent infections. Therefore, understanding *A. baumannii* biofilm formation, maturation, and dispersal is important for developing effective strategies to inhibit its growth and proliferation.

Colistin (CST) and tigecycline (TGC) are the last resort antibiotics used against a number of multidrug resistant bacteria^9^, although there have been reports of antibiotic resistance against these antibiotics worldwide^10,11^. Previous studies have suggested that a combination of TGC with CST, levofloxacin (LVX), amikacin (AMK), and imipenem (IPM) may be an effective therapy to synergistically prevent the emergence of resistance during treatment of MDRAB infections^12,13^. Moreover, Wang et al. reported the synergistic effects of the combinations of meropenem (MEPM), IPM, sulbactam (SBT), CST, and TGC on biofilm-embedded carbapenem-resistant *A. baumannii* and carbapenem-susceptible *A. baumannii* strains^14^. Meanwhile, other studies reported that no synergistic effects of CST and TGC were observed in *A. baumannii* clinical isolates^15,16^. Therefore, the combined effects of antibiotics on *A. baumannii* may depend on bacterial strains. Additionally, we have reported that clinical isolates of MDRAB showed different degrees of biofilm formation in the presence of sub-minimum inhibitory concentrations (sub-MICs) of CST and TGC^17^, suggesting that *A. baumannii* is emerging as a highly pathogenic bacterium and that the characteristics of *A. baumannii* vary under different environmental stress conditions, such as in the presence of multiple antimicrobial agents. Therefore, in this study, we focused on the combined effects of CST and TGC on MDRAB biofilms, that is, the inhibitory and bactericidal effects of these antibiotics on biofilm-dispersed and biofilm-embedded MDRAB. Furthermore, we analyzed the combined effects of CST and TGC on biofilms formed by MDRAB.

## Results

### Inhibitory and bactericidal effects of CST and TGC on biofilm-dispersed and biofilm-embedded MDRAB

We have previously reported the MICs of CST and TGC for MDRAB strains R1 and R2 in a previous study^17^. The MICs of CST on MDRAB strains R1 and R2 were at 2 μg/mL, and those of TGC were at 0.5 μg/mL, respectively (Table 1). We analyzed the bactericidal concentrations of CST and TGC on planktonic cells of MDRAB strains R1 and R2. The minimum bactericidal concentration (MBC) of CST on these cells was 8 μg/mL (i.e., a 4-fold increase in MIC) (Table 1). Meanwhile, the MBCs of TGC on these cells were > 256 and 256 μg/mL for R1 and R2, respectively (i.e., > 512 and 512-fold MIC increase, respectively) (Table 1). We further analyzed the inhibitory and bactericidal concentrations of CST and TGC on biofilm-dispersed MDRAB. Bacterial growth of biofilm-dispersed MDRAB strains R1 and R2 was inhibited with CST at 16 and 32 μg/mL (i.e., 8- and 16-fold MIC increase), and inhibited with TGC at 4 μg/mL, respectively (i.e., 8-fold MIC increase, respectively) (Table 1). The MBCs of CST on these cells were 32 μg/mL for R1 and R2, respectively (i.e., 16-fold MIC increase, respectively) (Table 1). Meanwhile, the MBCs of TGC on these cells were > 256 μg/mL for R1 and R2, respectively (i.e., > 512-fold MIC increase, respectively) (Table 1). Additionally, we analyzed the bactericidal concentrations of CST and TGC on biofilm-embedded MDRAB (i.e., minimum biofilm eradication concentrations: MBECs). The MBECs of CST on MDRAB strains R1 and R2 were 16 and 32 μg/mL, respectively (i.e., 8- and 16-fold MIC increase, respectively). Meanwhile, TGC had no bactericidal effects on MDRAB cells in strains R1 and R2, even at a concentration of 256 μg/mL (i.e., > 512-fold MIC increase) (Table 1). These results suggest that CST at 32 μg/mL has antibiotic effects not only for biofilm-dispersed but also for biofilm-embedded MDRAB, whereas TGC is an effective antibiotic for the inhibition of bacterial growth of biofilm-dispersed but not biofilm-embedded MDRAB.

**Table 1:** MIC and MBC of CST or TGC alone on planktonic and biofilm-dispersed MDRAB, and MBEC of CST or TGC alone on biofilm-embedded MDRAB.

Antibiotics	MDRAB	Planktonic cells		Biofilm-dispersed cells		Biofilm-embedded cells
		MIC	MBC	MIC	MBC	MBEC
**Concentration of antibiotics (μg/mL)**						
CST	R1	2	8	16	32	16
	R2	2	8	32	32	32
TGC	R1	0.5	> 256	4	> 256	> 256
	R2	0.5	256	4	> 256	> 256
**Fold increase (vs. MIC of planktonic cells)**						
CST	R1	1	4	8	16	8
	R2	1	4	16	16	16
TGC	R1	1	> 512	8	> 512	> 512
	R2	1	512	8	> 512	> 512

*MIC* minimum inhibitory concentration, *MBC* minimum bactericidal concentration, *MBEC* minimum biofilm eradication concentration.

### Inhibitory effects of the combinations of CST and TGC on the bacterial growth of biofilm-dispersed MDRAB

We analyzed the inhibitory effects of combinations of CST and TGC on the growth of biofilm-dispersed MDRAB. The growth of biofilm-dispersed MDRAB strain R1 was inhibited with 16 μg/mL of CST and 4 μg/mL of TGC (Tables 1 and 2). However, the inhibitory effects were slightly attenuated with the combination of CST at 16 μg/mL and TGC at 0.25–1 μg/mL (Table 2). Additionally, the inhibitory effect of CST at 8 μg/mL was significantly attenuated with the combination of TGC at 0.5 μg/mL (Table 2). Bacterial growth of biofilm-dispersed MDRAB strain R2 was inhibited with 32 μg/mL of CST and 4 μg/mL of TGC, respectively (Tables 1 and 3). However, the inhibitory effects were slightly attenuated by the combination of CST at 8 and 16 μg/mL and TGC at 0.25–1 μg/mL (Table 3). These results suggest that the inhibitory effects of CST on the bacterial growth of biofilm-dispersed MDRAB were attenuated when used in combination with low concentrations of TGC.

**Table 2:**
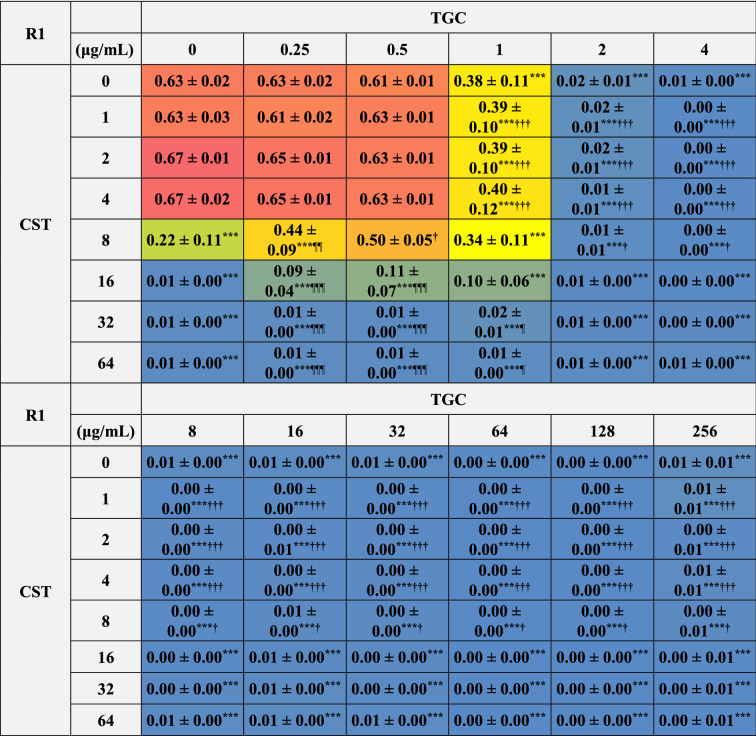
Bacterial growth of biofilm-dispersed MDRAB (R1) in the presence of CST and TGC, either alone or in combination.

Numeric data (A_595_) are shown as the mean ± SEM (n = 6) obtained from six independent experiments. ***P < 0.001; **P < 0.01; *P < 0.05, no treatment versus treatment with antibiotics; One-way ANOVA. ^**†††**^*P* < 0.001; ^**††**^*P* < 0.01; ^**†**^*P* < 0.05, treatment with CST alone versus treatment with CST in combination with TGC; One-way ANOVA. ^¶¶¶^*P* < 0.001; ^¶¶^*P* < 0.01; ^¶^*P* < 0.05, treatment with TGC alone versus treatment with TGC in combination with CST; One-way ANOVA. The colors indicate the levels of bacterial growth. Blue indicates no bacterial growth.

**Table 3:**
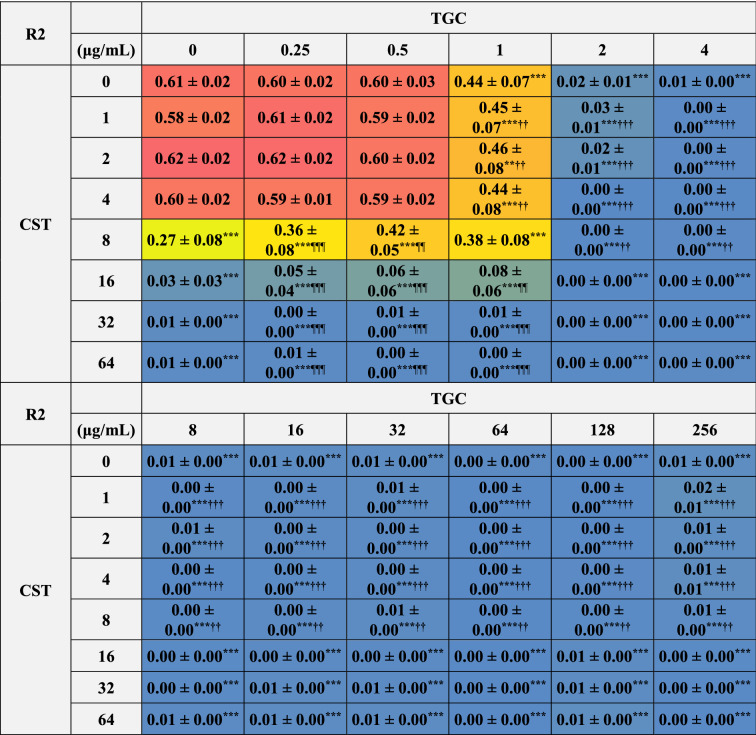
Bacterial growth of biofilm-dispersed cells of MDRAB (R2) in the presence of CST and TGC, either alone or in combination.

Numeric data (A_595_) are shown as the mean ± SEM (n = 6) obtained from six independent experiments. ***P < 0.001; **P < 0.01; *P < 0.05, no treatment versus treatment with antibiotics; One-way ANOVA. ^**†††**^*P* < 0.001; ^**††**^*P* < 0.01; ^**†**^*P* < 0.05, treatment with CST alone versus treatment with CST in combination with TGC; One-way ANOVA. ^¶¶¶^*P* < 0.001; ^¶¶^ *P* < 0.01; ^¶^*P* < 0.05, treatment with TGC alone versus treatment with TGC in combination with CST; One-way ANOVA. The colors indicate the levels of bacterial growth. Blue indicates no bacterial growth.

### Bactericidal effects of combinations of CST and TGC on biofilm-dispersed MDRAB

We further analyzed the bactericidal effects of combinations of CST and TGC on biofilm-dispersed MDRAB. The MBC of CST on biofilm-dispersed MDRAB strain R1 was 32 μg/mL, whereas TGC had no bactericidal effect even with treatment at 256 μg/mL (Tables 1 and 4). Meanwhile, the synergistic bactericidal effect of CST at 16 μg/mL in combination with TGC at > 1 μg/mL were observed in these cells (Table 4). However, the bactericidal effects of CST at 16 and 32 μg/mL were attenuated when used in combination with TGC at < 1 μg/mL (Table 4). The MBC of CST on biofilm-dispersed MDRAB strain R2 was 32 μg/mL, whereas that of TGC on these cells was > 256 μg/mL (Tables 1 and 5). Meanwhile, the synergistic bactericidal effects of CST at 16 μg/mL in combination with TGC at > 1 μg/mL were observed in these cells (Table 5). However, the bactericidal effects of CST at 16 and 32 μg/mL were attenuated when used in combination with 0.5 μg/mL of TGC (Table 5). These results suggest that the CST-TGC combination exhibits synergistic bactericidal effects on biofilm-dispersed MDRAB; however, the bactericidal effects of CST on these cells are attenuated when used in combination with low concentrations of TGC.

**Table 4:**
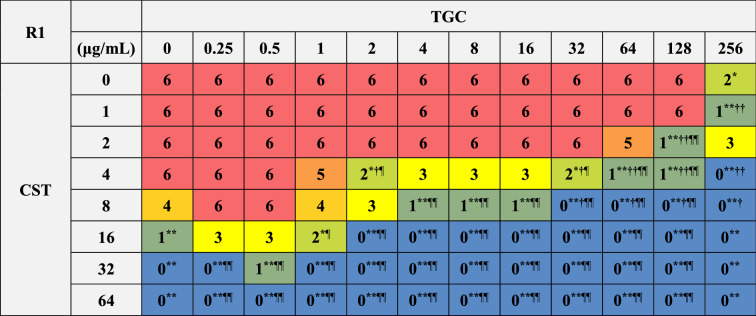
Colony detection from surviving biofilm-dispersed cells of MDRAB (R1) after treatments with CST and TGC, either alone or in combination.

Numeric data are shown as the number of colonies detected on the LB agar plates (n = 6) obtained from six independent experiments. **P < 0.01; *P < 0.05, no treatment versus treatment with antibiotics; Fisher's exact test. ^**††**^*P* < 0.01; ^**†**^*P* < 0.05, treatment with CST alone versus treatment with CST in combination with TGC; Fisher's exact test. ^¶¶^*P* < 0.01; ^¶^*P* < 0.05, treatment with TGC alone versus treatment with TGC in combination with CST; Fisher's exact test. Red color indicates colonies were detected in all six experiments, orange and light orange color mean colonies were detected in five and four out of six experiments, yellow color means colonies were detected in three out of six experiments, light green and green color mean colonies were detected in two and one of six experiments, blue color means no colonies were detected in any experiment.

**Table 5:**
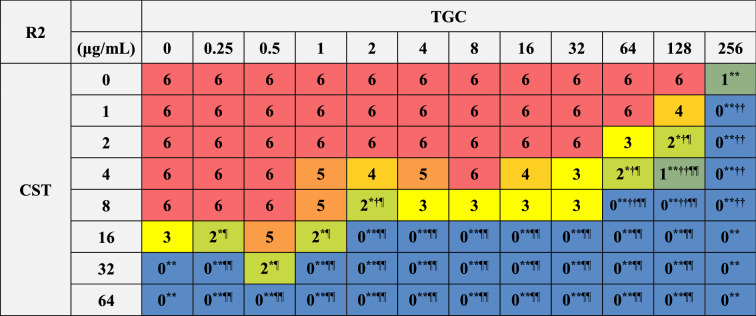
Colony detection from surviving biofilm-dispersed cells of MDRAB (R2) after treatments with CST and TGC, either alone or in combination.

Numeric data are shown as the number of colonies detected on the LB agar plates (n = 6) obtained from six independent experiments. **P < 0.01; *P < 0.05, no treatment versus treatment with antibiotics; Fisher's exact test. ^**††**^*P* < 0.01; ^**†**^*P* < 0.05, treatment with CST alone versus treatment with CST in combination with TGC; Fisher's exact test. ^¶¶^*P* < 0.01; ^¶^*P* < 0.05, treatment with TGC alone versus treatment with TGC in combination with CST; Fisher's exact test. Red color indicates colonies were detected in all six experiments, orange and light orange color mean colonies were detected in five and four out of six experiments, yellow color means colonies were detected in three out of six experiments, light green and green color mean colonies were detected in two and one of six experiments, blue color means no colonies were detected in any experiment.

### Bactericidal effects of combinations of CST and TGC on biofilm-embedded MDRAB

We analyzed the bactericidal effects of the combinations of CST and TGC on biofilm-embedded MDRAB, that is, the biofilm eradication effects of these antibiotics. The MBEC of CST on biofilm-embedded MDRAB strain R1 was 16 μg/mL, whereas no bactericidal effect was observed even when 256 μg/mL of TGC was used on these cells (Tables 1 and 6). Meanwhile, synergistic bactericidal effects of CST at 8 μg/mL on these cells were observed when used in combination with 128 μg/mL of TGC (Table 6). However, the bactericidal effects of CST at 8–32 μg/mL were attenuated when used in combination with < 32 μg/mL of TGC (Table 6). Similarly, the MBEC of CST on biofilm-embedded MDRAB strain R2 was 32 μg/mL, whereas no bactericidal effect was observed even with treatment of 256 μg/mL of TGC on these cells (Tables 1 and 7). Meanwhile, a synergistic bactericidal effect of CST at 16 μg/mL on these cells was observed when used in combination with 32 μg/mL of TGC (Table 7). However, the bactericidal effects of CST at 16 μg/mL were attenuated when used in combination with 0.5–2 μg/mL of TGC (Table 7). Additionally, the inhibitory effects of TGC at 128 μg/mL were attenuated when used in combination with 2 μg/mL of CST on biofilm-embedded MDRAB strains R1and R2 (Tables 6 and 7). These results suggest that the combination of CST and a high concentration of TGC, except for when used in combination with 2 μg/mL of CST and 128 μg/mL of TGC, exhibits synergistic bactericidal effects on biofilm-embedded MDRAB; however, the bactericidal effects of CST on these cells were attenuated when used in combination with low concentrations of TGC.

**Table 6:**
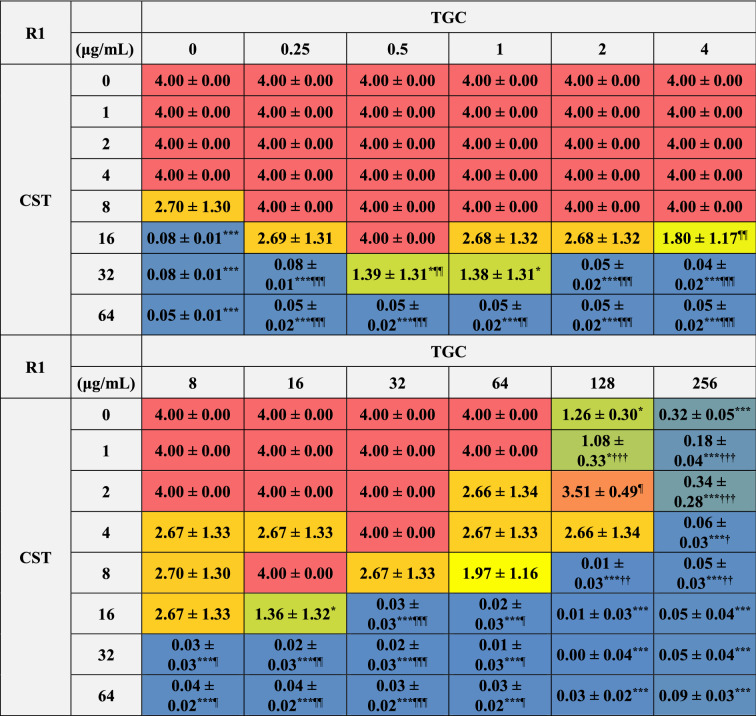
Bacterial growth of biofilm-embedded MDRAB (R1) after treatment with CST and TGC, either alone or in combination.

Numeric data (A_450_) are shown as the mean ± SEM (n = 3) obtained from three independent experiments. The range of detection was < 4.00. ***P < 0.001; **P < 0.01; *P < 0.05, no treatment versus treatment with antibiotics; One-way ANOVA. ^**†††**^*P* < 0.001; ^**††**^*P* < 0.01; ^**†**^*P* < 0.05, treatment with CST alone versus treatment with CST in combination with TGC; One-way ANOVA. ^¶¶¶^*P* < 0.001; ^¶¶^*P* < 0.01; ^¶^*P* < 0.05, treatment with TGC alone versus treatment with TGC in combination with CST; One-way ANOVA. The colors indicate the levels of bacterial growth. Blue indicates no bacterial growth.

**Table 7:**
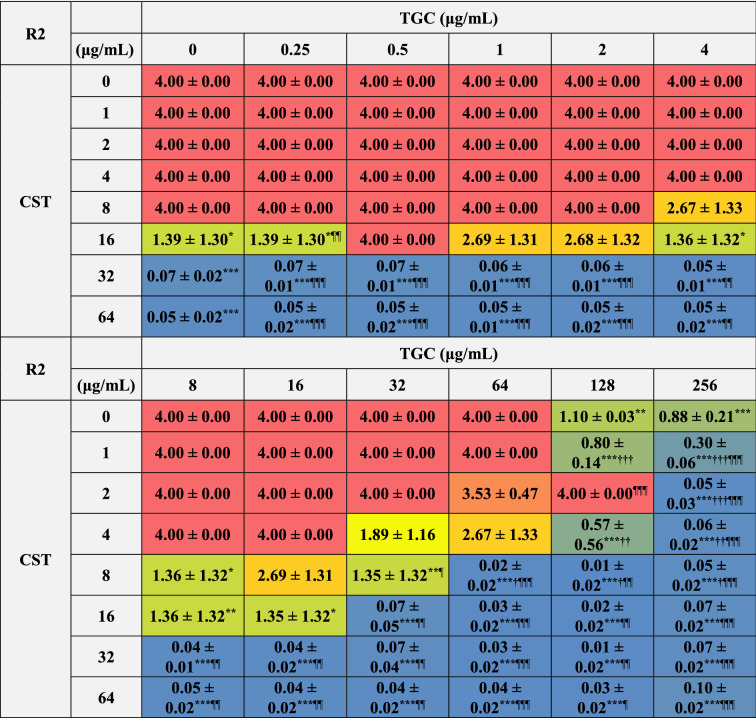
Bacterial growth of biofilm-embedded MDRAB (R2) after treatment with CST and TGC, either alone or in combination.

Numeric data (A_450_) are shown as the mean ± SEM (n = 3) obtained from three independent experiments. The range of detection was < 4.00. ***P < 0.001; **P < 0.01; *P < 0.05, no treatment versus treatment with antibiotics; One-way ANOVA. ^**†††**^*P* < 0.001; ^**††**^*P* < 0.01; ^**†**^*P* < 0.05, treatment with CST alone versus treatment with CST in combination with TGC; One-way ANOVA. ^¶¶¶^*P* < 0.001; ^¶¶^*P* < 0.01; ^¶^*P* < 0.05, treatment with TGC alone versus treatment with TGC in combination with CST; One-way ANOVA. The colors indicate the levels of bacterial growth. Blue indicates no bacterial growth.

### Effects of CST and TGC used alone or in combination on biofilm formation of MDRAB

We further analyzed whether CST and TGC affected the biofilms formed by MDRAB. Biofilms formed by MDRAB of strains R1 and R2 decreased depending on the concentration of CST, whereas they decreased slightly depending on the concentration of TGC (Tables 8 and 9). However, the biofilms formed by MDRAB strain R1 significantly increased with the combination of 1 μg/mL of CST and 2 μg/mL of TGC (Table 8). Meanwhile, biofilms formed by MDRAB in strain R2 increased with the combination of 1–2 μg/mL of CST and 2–4 μg/mL of TGC (Table 9). Additionally, there was no synergistic disruptive effect of the combination of CST and TGC on these biofilms (Tables 8 and 9).

**Table 8:**
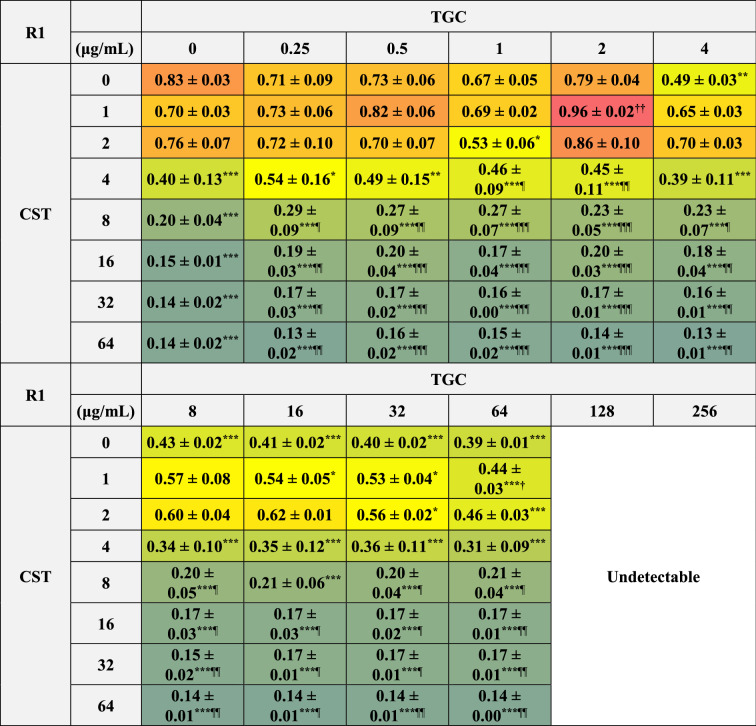
Biofilm formation of MDRAB (R1) after treatment with CST and TGC, either alone or in combination.

Numeric data (A_595_) are shown as the mean ± SEM (n = 3) obtained from three independent experiments. ***P < 0.001; **P < 0.01; *P < 0.05, no treatment versus treatment with antibiotics; One-way ANOVA. ^**†††**^*P* < 0.001; ^**††**^*P* < 0.01; ^**†**^*P* < 0.05, treatment with CST alone versus treatment with CST in combination with TGC; One-way ANOVA. ^¶¶¶^*P* < 0.001; ^¶¶^*P* < 0.01; ^¶^*P* < 0.05, treatment with TGC alone versus treatment with TGC in combination with CST; One-way ANOVA. Colors indicate the level of biofilm formation. A_595_ at TGC concentrations of 128 and 256 μg/mL were undetectable because the reconstituted solution was yellow to orange in color.

**Table 9:**
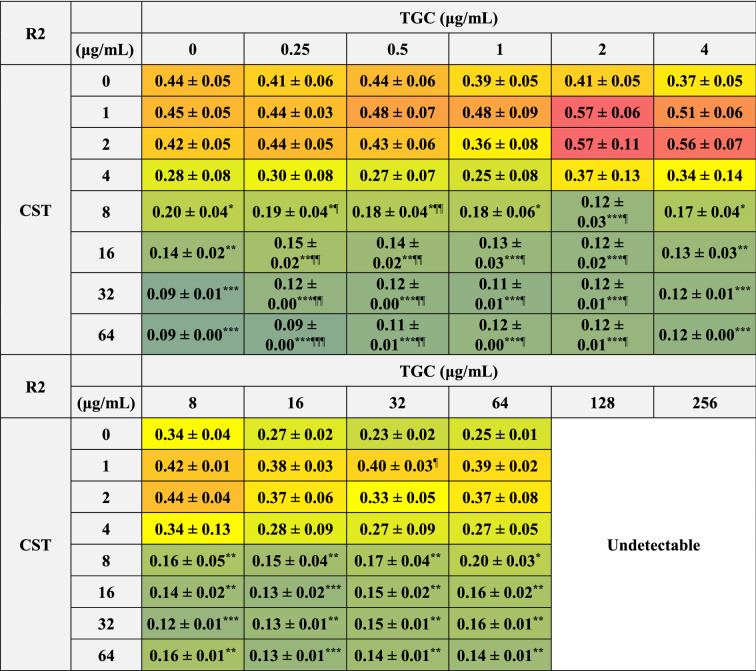
Biofilm formation of MDRAB (R2) after treatment with CST and TGC, either alone or in combination.

Numeric data (A_595_) are shown as the mean ± SEM (n = 3) obtained from three independent experiments. ***P < 0.001; **P < 0.01; *P < 0.05, no treatment versus treatment with antibiotics; One-way ANOVA. ^**†††**^*P* < 0.001; ^**††**^*P* < 0.01; ^**†**^*P* < 0.05, treatment with CST alone versus treatment with CST in combination with TGC; One-way ANOVA. ^¶¶¶^*P* < 0.001; ^¶¶^*P* < 0.01; ^¶^*P* < 0.05, treatment with TGC alone versus treatment with TGC in combination with CST; One-way ANOVA. Colors indicate the level of biofilm formation. A_595_ at TGC concentrations of 128 and 256 μg/mL were undetectable because the reconstituted solution was yellow to orange in color.

## Discussion

*Acinetobacter baumannii* has recently emerged as a major nosocomial pathogen^1,2^, and an increase in outbreaks of MDRAB worldwide is becoming a cause of concern^3,4^. Additionally, it is possible that *A. baumannii* causes biofilm-associated infections, such as bacteremia, via patient indwelling devices^5,7,18,19^ because *A. baumannii* is capable of forming biofilms^2,5,6^. Khazaal et al*.* suggested that biofilms formed in endotracheal tubes lead to the development of ventilator-associated pneumonia (VAP)^20^. Moreover, catheter-related urinary or bloodstream infections are caused by biofilm-forming strains^21^. Therefore, biofilm formation is thought to be an important pathogenic feature, especially in relation to intravascular line infections and VAP, so treatment strategies for inhibition of biofilm formation may lead to a reduced risk of severe *A. baumannii* infection.

CST and TGC remain the only effective antibiotics for the treatment of MDRAB^9^. CST is an old antibiotic member of the polymyxin family, which are membrane-active peptides with bactericidal capabilities that can disrupt the outer bacterial cell membrane^22^. It is currently used as a last-resort antibiotic for the treatment of multidrug-resistant gram-negative bacterial infections^23^. Meanwhile, TGC is a member of the glycylcycline class of semisynthetic antimicrobial agents developed for the treatment of polymicrobial infections caused by multidrug-resistant gram-positive and gram-negative bacteria and acts as a bacteriostatic agent against these bacteria^24^. In fact, the bactericidal effects of CST were observed on biofilm-dispersed and biofilm-embedded MDRAB, whereas TGC had no bactericidal effect on these cells. These results reflect the reasonable action of these antibiotics. Furthermore, we demonstrated that the combination of CST and TGC exhibited synergistic bactericidal effects on biofilm-dispersed and biofilm-embedded MDRAB. However, although the synergistic effects of CST on these cells increased when used in combination with high concentrations of TGC, they were attenuated when used in combination with low concentrations of TGC. It remains unclear why the antibiotic activity of CST on MDRAB is attenuated when used in combination with low concentrations of TGC. Efflux pumps play an important role in antimicrobial resistance and virulence for *A. baumannii*^25^. Lin et al. reported that *EmrB*-deleted mutant of *A. baumannii* was more susceptible to CST, suggesting that EmrAB pump systems in *A. baumannii* contribute to the resistance to CST^26^. Additionally, Cheng et al. reported that the expression of efflux pumps including EmrAB in *A. baumannii* upregulated under the pressure of TGC at a low concentration^27^. With considering these results, we speculate that bactericidal effects of CST were attenuated when used in combination with low concentrations of TGC because of EmrAB efflux pumps upregulated by TGC at low concentrations. Additionally, these results suggest that the antibiotic resistance characteristics of *A. baumannii* vary under different environmental stress conditions (e.g., changes in antibiotic concentrations, combination of various antibiotics, etc.). We previously reported that clinical isolates of MDRAB showed different degrees of biofilm formation in the presence of sub-MICs of CST and TGC^17^. Moreover, we demonstrated that biofilm formation by MDRAB increased even more upon treatment with CST in combination with TGC at concentrations less than the MBC, suggesting that the characteristics of *A. baumannii* vary in the presence of antibiotics at low concentrations. We have reported that the number of biofilm cells was positively and significantly correlated with the mRNA levels of genes encoding efflux pumps (*adeB* and *adeG*) and autoinducer synthase (*abaI*), which are biofilm-related genes in MDRAB strain R2 in the presence of CST^17^. Additionally, several reports demonstrated that mRNA levels of genes encoding efflux pumps, porins, virulence factors, and biofilm-related genes in *A. baumannii* were altered in the presence of various antibiotics at sub-MICs^28,29^. Therefore, we speculate that the expression of biofilm-related genes in biofilm-embedded MDRAB is altered in the presence of CST and TGC because these cells are exposed to low concentrations of antibiotics that slightly infiltrate into biofilms. Therefore, exposure to a lethal dose of bactericidal concentrations of CST and TGC in patients is necessary for the treatment of *A. baumannii* infection.

In summary, we demonstrated the combined effects of CST and TGC on biofilm-dispersed MDRAB, biofilm-embedded MDRAB, and biofilms formed by MDRAB. Since MDRAB clinical isolates showed different characteristics upon treatment with antibiotics, further studies are required to understand the mechanism of the biofilm-related characteristics of *A. baumannii* in the presence of antibiotics.

## Materials and methods

All methods were carried out in accordance with relevant guidelines and regulations.

### Bacterial strains and growth conditions

R1 and R2 strains of *A. baumannii* were isolated from the Teikyo University Hospital during an outbreak that occurred around 2010. The bacteria were isolated on CHROMagar™ *Acinetobacter* and incubated for 24 h at 37 °C. The R1 strain was isolated from the sputum of a patient with interstitial pneumonia. The R2 strain was isolated from a urine sample of a patient with malignant lymphoma and pneumonia. The isolates were streaked onto blood agar plates and cultivated for 24 h to obtain monoclonal colonies and were identified as *A. baumannii* through DNA sequencing of the partial RNA polymerase β-subunit (*rpoB*) gene^30^. Additionally, the isolates were confirmed to be non-clonal via pulsed-field gel electrophoresis (data not shown). After identification, the isolates were stored in glycerol stocks at − 80 °C at the Department of Microbiology & Immunology, Teikyo University School of Medicine. Antimicrobial susceptibility testing was performed using two strains of *A. baumannii* based on the MICs of IPM, AMK, and ciprofloxacin (CPFX). Against these two strains, the MICs of IPM, AMK, and CPFX were > 8, > 32, and > 2 mg/L, respectively. Thus, these strains were identified as MDRAB strains. The MIC of CST against MDRAB in strains R1 and R2 was determined to be 2 mg/L^17^. The MIC of TGC against these strains was found to be 0.5 mg/L^17^. These bacteria were cultured on Luria–Bertani (LB) agar plates (Becton, Dickinson and Company, MD, USA) for 16 h at 37 °C. Thereafter, the bacteria were suspended in LB broth at a concentration of OD_595_ = 0.1, then the concentration was adjusted via optical density (OD) measurements at 595 nm. The bacterial suspensions obtained were used for biofilm formation.

### Determination of inhibitory and bactericidal effects of antibiotics on biofilm-dispersed MDRAB, biofilm-embedded MDRAB, and biofilms formed by MDRAB

To analyze the combined effects of CST and TGC on biofilm-dispersed MDRAB, biofilm-embedded MDRAB, and biofilms formed by MDRAB, bacterial strains were added to 96-well microtiter plates containing the Biofilm Formation Assay Kit or Biofilm Viability Assay Kit (DOJINDO, Kumamoto, Japan)^31^. Then, the plate was covered with a 96-pin microtiter plate lid and incubated for 24 h at 37 °C. After incubation, the 96-pin lid with established biofilms was washed three times with sterile PBS and then placed onto a fresh 96-well microtiter plate with each well containing LB with various concentrations of antibiotics. After incubation for 24 h at 37 °C, to analyze the inhibitory effects of antibiotics on biofilm-dispersed MDRAB, the 96-pin lid was removed and the culture broth was measured at A_595_. To analyze the bactericidal effects of antibiotics on biofilm-dispersed MDRAB, 5 μL of culture broth was plated onto LB agar, and the presence of viable cells was confirmed through the growth from the bacterial suspension after 24 h of incubation at 37 °C. To analyze the number of biofilm cells, the 96-pin lid was washed three times with sterile PBS and then placed onto a fresh 96-well microtiter plate with each well containing 0.1% crystal violet solution. After staining for 30 min, the 96-pin lid with stained biofilms was washed three times with sterile PBS to remove unreacted crystal violet. Then, the 96-pin lid was placed onto a fresh 96-well microtiter plate with each well containing 99.5% ethanol and extracted for 15 min. The amount of crystal violet extracted was measured using A_595_. The viability of biofilm-embedded MDRAB was determined using the Biofilm Viability Assay Kit (DOJINDO) according to the manufacturer’s protocol. After the incubation of biofilms in the presence of antibiotics, the 96-pin lid was washed three times with sterile PBS to remove planktonic and nonattached cells and then placed onto a fresh 96-well microtiter plate with each well containing LB broth with WST solution. After incubation for 24 h, the 96-pin lid was removed, and the LB broth was measured at A_450_.
